# Combined LDL and VLDL Electronegativity Correlates with Coronary Heart Disease Risk in Asymptomatic Individuals

**DOI:** 10.3390/jcm8081193

**Published:** 2019-08-09

**Authors:** Ming-Yi Shen, Jing-Fang Hsu, Fang-Yu Chen, Jonathan Lu, Chia-Ming Chang, Mohammad Madjid, Juliette Dean, Richard A. F. Dixon, Steven Shayani, Tzu-Chieh Chou, Chu-Huang Chen

**Affiliations:** 1 Graduate Institute of Biomedical Sciences, China Medical University, Taichung 404, Taiwan; 2 Department of Medical Research, China Medical University Hospital, Taichung 404, Taiwan; 3 Department of Nursing, Asia University, Taichung 404, Taiwan; 4 National Institute of Environmental Health Sciences, National Health Research Institutes, Miaoli 360, Taiwan; 5 Vascular and Medicinal Research, Texas Heart Institute, Houston, TX 77030, USA; 6 InVitro Cell Research, LLC, Englewood Cliffs, NJ 07631, USA; 7 Cardiovascular Research Laboratory, China Medical University Hospital, Taichung 404, Taiwan; 8 Cardiovascular Division, Department of Internal Medicine, McGovern Medical School, The University of Texas Health Sciences Center at Houston, Houston, TX 77030, USA; 9 Electrophysiology Clinical Research & Innovations, Texas Heart Institute, Houston, TX 77030, USA; 10 Center for Clinical Research, Texas Heart Institute, Houston, TX 77030, USA; 11 Department of Molecular Cardiology, Texas Heart Institute, Houston, TX 77030, USA; 12 Mount Sinai Medical Center, Mineola, FL 33140, USA; 13 New York Heart Research Foundation, Mineola, NY 11501, USA; 14 Department of Public Health, China Medical University, Taichung 404, Taiwan; 15 Department of Health Risk Management, China Medical University, Taichung 404, Taiwan

**Keywords:** electronegativity, human plasma LDL, L5, human plasma VLDL, V5, hard CHD risk, ApoE^−/−^ mice, aortic lipid accumulation, cellular senescence

## Abstract

The most electronegative constituents of human plasma LDL (i.e., L5) and VLDL (i.e., V5) are highly atherogenic. We determined whether the combined electronegativity of L5 and V5 (i.e., L5 + V5) plays a role in coronary heart disease (CHD). In 33 asymptomatic individuals (ages 32–64), 10-year hard CHD risk correlated with age (r = 0.42, *p* = 0.01). However, in age-adjusted analyses, 10-year hard CHD risk correlated with L5 + V5 plasma concentration (r = 0.43, *p* = 0.01) but not age (*p* = 0.74). L5 + V5 plasma concentration was significantly greater in the group with high CHD risk (39.4 ± 22.0 mg/dL; *n* = 17) than in the group with low CHD risk (16.9 ± 14.8 mg/dL; *n* = 16; *p* = 0.01). In cultured human aortic endothelial cells, L5 + V5 treatment induced significantly more senescence-associated–β-Gal activity than did equal concentrations of L1 + V1 (*n* = 4, *p* < 0.001). To evaluate the in vivo relevance of these findings, we fed ApoE^−/−^ and wild-type mice with a high-fat diet and found that plasma LDL, VLDL, and LDL + VLDL from ApoE^−/−^ mice exhibited significantly greater electrophoretic mobility than did wild-type counterparts (*n* = 6, *p* < 0.01). The increased electronegativity of LDL and VLDL in ApoE^−/−^ mice was accompanied by increased aortic lipid accumulation and cellular senescence (*n* = 6, *p* < 0.05). Clinical trials are warranted to test the predictive value of L5 + V5 concentration in patients with CHD.

## 1. Introduction

Cardiovascular disease (CVD) remains the leading cause of death in the United States and around the world [1,2,3]. In the Global Burden of Disease Study, it was estimated that 31% of all deaths worldwide (i.e., 57.74 million deaths) were caused by CVD in 2015 [4]. In the United States specifically, 840,678 CVD deaths were registered in 2016, a number that increased from 836,546 deaths in 2015 [3]. Coronary heart disease (CHD) is the major cause of CVD deaths, accounting for 375,295 (47.7%) of all 786,641 deaths in the United States in 2011 [1]. An estimated one-half of all middle-aged men and one-third of middle-aged women in the United States will develop some manifestation of CHD [5]. CHD incidence and death rates are strongly correlated with age. The prevalence of CHD increases with each 20-year incremental age increase in both men (ages 20–39, 0.6%; ages 40–59, 6.3%; ages 60–79, 19.9%; and ages 80+, 32.2%) and women (ages 20–39, 0.6%; ages 40–59, 6.3%; ages 60–79, 9.7%; and ages 80+, 18.8%) in the United States [1]. 

Epidemiologic studies have shown that age is the predominant risk factor for atherosclerotic CVD [6]. Because aging involves various biologic phenomena, attributing age-related changes of the vasculature or an organism to a specific molecule or pathway has been challenging. In the 1960s, Hayflick and colleagues first hypothesized that cellular senescence is associated with aging [7,8]. Minamino and colleagues [9] later used cellular senescence as a model of aging in vivo to show that vascular cell senescence contributes to the pathogenesis of age-associated vascular diseases such as atherosclerosis. Senescent vascular cells accumulate in human atheroma tissues and exhibit various features of dysfunction [9]. Previous studies have indicated that L5, the most electronegative subfraction of LDL isolated by using ion-exchange chromatography, is the only LDL subfraction that can induce endothelial dysfunction and atherogenic responses in cultivated arteries and cultured vascular cells [10,11]. Recently, our group showed that naturally occurring L5 may promote mitochondrial free-radical production and activate the DNA damage response to induce premature senescence in the endothelium, subsequently leading to atherosclerosis [12,13,14]. Further evidence has shown that increased plasma L5 levels are associated with multiple CVD risk factors and CVD risk, suggesting that the LDL electronegativity index may be a novel predictor of CVD [15,16,17,18,19,20,21]. In addition to L5, we have shown that the most electronegative subfraction of VLDL isolated by using ion-exchange chromatography, called V5, is also highly cytotoxic [22]. However, evidence of a clinical correlation between negatively charged lipoproteins and CVD risk has been lacking. In this study, we assessed the combined adverse effects of the negatively charged apoB-containing lipoproteins L5 and V5 (i.e., L5 + V5) on CVD risk by exploring the relationship between hard CHD risk and the amount of L5 + V5 in blood plasma. In addition, we studied the senescence response in vessel endothelia to provide a biologic explanation of CHD that results from exposure to negatively charged lipoproteins.

## 2. Research Design and Methods

### 2.1. Study Participants

In this study, we enrolled 33 participants (age range, 30–74 years old) without CHD who had not received statin treatment or any other lipid-lowering therapy in the previous 3 months. All study participants were seen by cardiologists at the Texas Heart Institute (Houston, TX, USA) or at the New York Heart Research Foundation (New York, NY, USA). Written consent was provided by all participants before plasma collection. The study protocol was approved by the St. Luke’s Episcopal Hospital Institutional Review Board (Houston, TX, USA) and the New York Heart Research Foundation (Mineola, NY, USA). This study was conducted according to the principles in the Declaration of Helsinki.

### 2.2. Sample Collection

All study participants were instructed to fast before blood collection so that lipid concentrations would represent stable lipid levels. Venous blood samples (30 mL) were drawn from each participant by using BD VACUETTE^®^ EDTA Blood Tubes (Becton, Dickinson and Company, Franklin Lakes, NJ, USA) containing anti-coagulants. A questionnaire was administered to each participant that included a range of questions related to personal characteristics (e.g., age, sex) and lifestyle (e.g., tobacco usage, medical history). Information obtained from the questionnaire was used in the statistical analyses. The analysis of lipid levels and other biochemical parameters was performed in the Department of Laboratory Medicine at the Texas Heart Institute or at the New York Heart Research Foundation (accredited by the College of American Pathologists) according to standard operating procedures.

### 2.3. VLDL and LDL Isolation and Separation

Plasma samples were isolated from 33 participants. As described previously, plasma VLDL and LDL were isolated from patients with metabolic syndrome by using sequential potassium bromide density-gradient ultra-centrifugation between a density range of 1.006 and 1.063 g/mL. To prevent contamination and experimental oxidation, we added the following to plasma samples immediately after collection: protease inhibitor cocktail (Roche Diagnostics, Indianapolis, IN), 1% penicillin/streptomycin/neomycin mixture (Invitrogen, Carlsbad, CA), and 0.5 mM EDTA. VLDL and LDL samples were resolved into subfractions V1–V5 and L1–L5, respectively, with increasingly negative charge on UnoQ12 columns (BioRad, Hercules, CA) by using an ion-exchange fast-protein liquid chromatography system (FPLC, GE Healthcare, Chicago, IL), as described previously [10,22]. The columns were equilibrated with buffer A (0.02 M Tris–HCl, pH 8.0; 0.5 mM EDTA). Subfractions were eluted with a multi-step linear gradient of buffer B (1 M NaCl in buffer A) at a flow rate of 2 mL/min and were monitored at 280 nm. The LDL subfractions from patients with metabolic syndrome were separately concentrated by using Centriprep filters (YM-30; EMD Millipore Corp., Billerica, MA) and sterilized by passing through 0.22-μm filters. The protein concentration of the VLDL and LDL subfractions was measured by using the Lowry method [2,10,22].

### 2.4. Cell Culture and Cellular Senescence Assay

Primary human aortic endothelial cells (HAECs) were cultured in EGM2 medium (Lonza) for cell studies [23]. HAECs were cultured in 12-well plates until the cells reached 80% confluency before being used in cell experiments (*n* = 4 per group). To evaluate the effect of L5 + V5 on cellular senescence, HAECs were incubated continuously for 3 days with subapoptotic levels of L5 (30 µg/mL) and/or V5 (5 µg/mL), L1 (30 µg/mL) and/or V1 (5 µg/mL), or phosphate-buffered saline (PBS, lipoprotein-free control). Then, senescence was analyzed by using a Senescence-associated β-Galactosidase (SA-β-Gal) Staining Kit (Cell Signaling, Danvers, MA) according to the manufacturer’s instructions [24]. Senescent blue-stained cells were counted by using microscopy, and at least 30,000 cells were counted in 9 random fields to determine the percentage of SA-β-Gal–positive cells.

### 2.5. Mice

All mouse experiments were approved by the China Medical University Institutional Animal Care and Use Committee in accordance with the Guide for the Care and Use of Laboratory Animals published by US National Institutes of Health (NIH Publication No. 85-23, revised 1996). Two species of animals were used to determine the effects of electronegative VLDL and LDL on cellular senescence. Apolipoprotein E (ApoE) knockout mice (ApoE^−/−^ mice) were fed with normal diet and a high-fat diet (TestDiet 58Y1) for 15 weeks and were used to determine endogenous levels of electronegative VLDL and LDL. C57BL6/J wild-type (WT) mice were used as controls. 

### 2.6. Electrophoresis Assay 

The electrophoretic mobility of VLDL and LDL samples (2.5 μg in 9 μL) was analyzed by using gel electrophoresis in 0.7% agarose (90 mM Tris and 90 mM boric acid, pH 8.2) at 100 V for 1.4 h [11].

### 2.7. SA-β-Gal Staining and Oil Red O Staining in Aortas

Mice (*n* = 6 per group) were anesthetized and euthanized by cervical dislocation, and the descending thoracic aorta was removed. Senescence activity was analyzed by using the SA-β-Gal Staining kit (Cell Signaling) according to the manufacturer’s instructions [14]. Using images acquired with a Canon EOS 70D digital camera, we identified senescent cells marked by a blue color produced by the enzymatic reaction. Atherosclerotic plaques were visualized by using Oil Red O staining. Aortic arches were fixed with 4% paraformaldehyde and stained with Oil Red O (Sigma) for 1 h [11].

### 2.8. Statistical Analysis

All data are presented as a frequency for discrete responses and as the mean ± standard deviation for continuous responses. For all parameters examined in this study, the Shapiro-Wilk normality test was used to determine whether a random sample of values followed a normal distribution. To compare differences between 2 groups, a nonparametric Mann-Whitney test was used for continuous data, and a Chi-square test or Fisher exact test was used for binary data. The associations between L5 + V5 levels and other hard CHD risk factors, including age, body mass index (BMI), pulse pressure, fasting plasma glucose level, and total cholesterol level, were evaluated by using the Spearman rank correlation coefficient. Ten-year hard CVD risk was calculated by using the Framingham risk score (developed by the large epidemiologic Framingham Heart Study [25]). The hard CVD category included coronary death, myocardial infarction, and stroke. The predictors used to calculate the Framingham risk score included sex, age, blood pressure, information regarding the treatment of hypertension and diabetes mellitus, smoking status, BMI, total cholesterol levels, and high-density lipoprotein (HDL) levels [25,26]. A *p*-value of *p* < 0.05 was considered statistically significant. Statistical analyses were performed by using the Statistical Package for Social Science (version 19.0; SPSS Inc., Chicago, IL, USA) software system.

## 3. Results

### 3.1. Combined L5 + V5 Plasma Levels Are Associated with Hard CHD Risk

**Figure 1:**
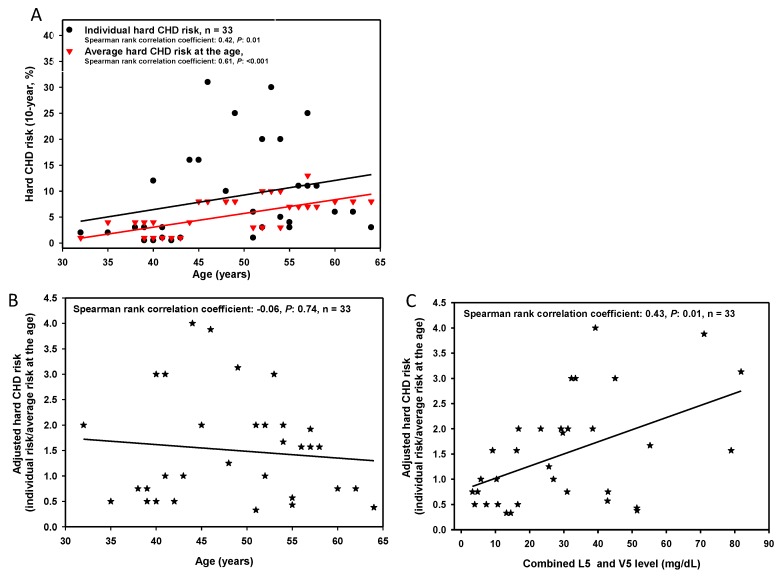
The relationship between combined L5 and V5 levels and coronary heart disease (CHD) risk according to the Framingham risk score. (**A**) Correlation between age and 10-year hard CHD (i.e., myocardial infarction, coronary death, or stroke) risk according to the Framingham risk score. (**B**) Correlation between age and adjusted hard CHD risk (i.e., hard CHD risk/average hard CHD risk at the age). The average hard CHD risk at the age is derived from the Framingham Heart Study [25] of a predominantly Caucasian population in Massachusetts, USA. (**C**) Correlation between the combined amount of L5 and V5 and adjusted hard CHD risk.

**Figure 2:**
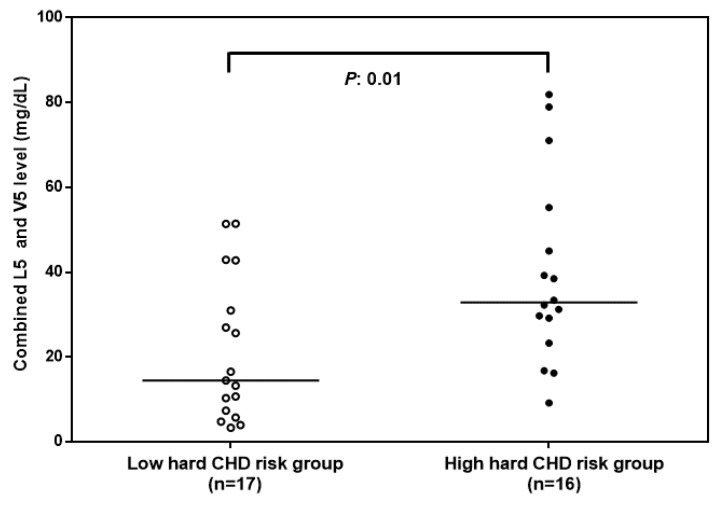
Combined electronegative VLDL and LDL levels in groups of study participants with high or low hard coronary heart disease (CHD) risk. Combined L5 and V5 levels were significantly higher in the high hard CHD risk group (adjusted hard CHD risk ≥ 1.5). The *p*-value was calculated by using the Mann-Whitney U test. Adjusted hard CHD risk was calculated as the hard CHD risk/average hard CHD risk at the age. The average hard CHD risk at the age was derived from the Framingham heart study [25] of a predominantly Caucasian population. The solid line indicates the median value for each group.

In our study population (*n* = 33), the 10-year hard CHD risk was significantly associated with age (r = 0.42, *p* < 0.01). In addition, the adjusted 10-year hard CHD risk was significantly associated with the amount of L5 + V5 (Figure 1). Table 1 shows a comparison of the basic characteristics of high (*n* = 16) and low (*n* = 17) hard CHD risk groups. Interestingly, in the group with high hard CHD risk, levels of L5 + V5 were higher than those in the group with low hard CHD risk (*p* = 0.01, Figure 2). Our results indicate that combined L5 + V5 levels contribute to hard CHD risk.

### 3.2. Combined L5 + V5 Plasma Levels Are Associated with Age and Select Hard CHD Risk Factors

**Figure 3:**
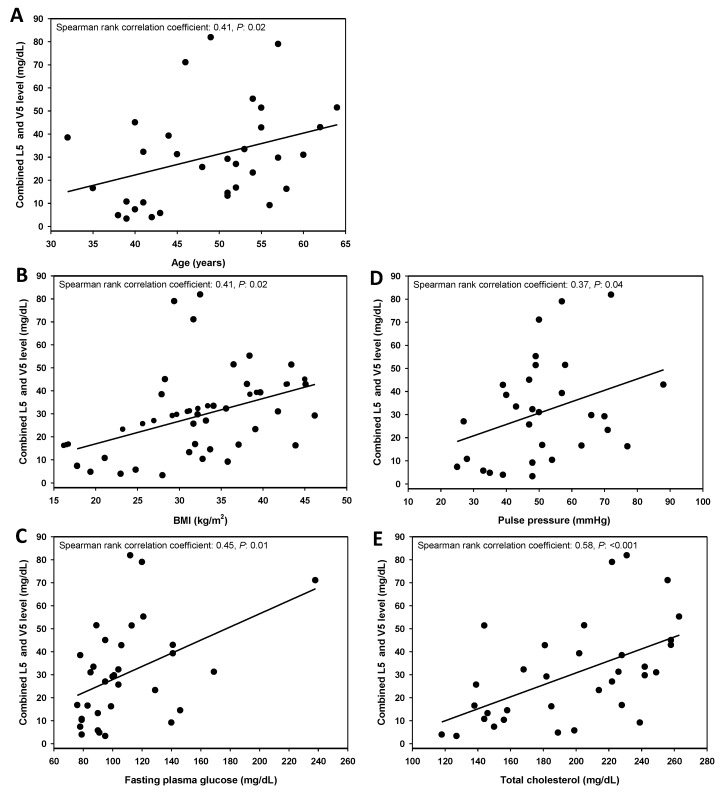
Correlation between combined L5 and V5 levels and select coronary heart disease (CHD) risk factors (*n* = 33). The combined L5 and V5 values were plotted against age (**A**), body mass index (BMI) (**B**), fasting plasma glucose level (**C**), pulse pressure (**D**), and total cholesterol level (**E**).

We next analyzed the correlation between combined L5 + V5 levels and hard CHD risk factors (Figure 3). Combined L5 + V5 levels increased with age, indicating that the senescence response may correlate with L5 + V5 levels. In addition, other hard CHD risk factors including BMI, pulse pressure, fasting plasma glucose, and total cholesterol were significantly correlated with L5 + V5 levels, indicating that L5 + V5 may increase hard CHD risk through their effects on vessel endothelial senescence, lipoprotein content, and lipoprotein constituents.

### 3.3. L5 + V5 Promote Cellular Senescence in HAECs

**Figure 4:**
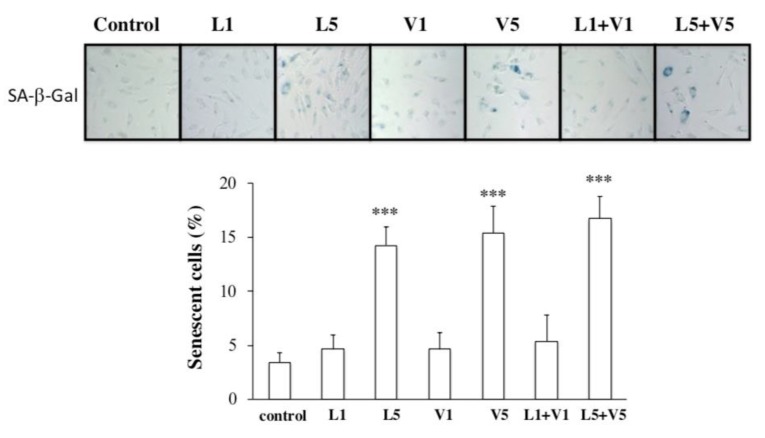
The combined effect of L5 and V5 on cellular senescence in cultured human aortic endothelial cells (HAECs). After treatment with phosphate-buffered saline (PBS; control), L1 (30 µg/mL), L5 (30 µg/mL), V1 (5 µg/mL), V5 (5 µg/mL), or the combination of L1 + V1 or L5 + V5 for 72 h, HAECs were stained with senescence-associated β-galactosidase (SA-β-Gal, blue), and the number of positive-stained cells was quantified. For the quantitative analyses, *n* = 4 per group. *** *p* < 0.001 vs. PBS-treated control.

To examine the effect of L5 + V5 on endothelial cell senescence, we treated HAECs in vitro with L5 + V5 or L1 + V1 isolated from the plasma of patients with high CHD risk. The combination of L5 (30 µg/mL) + V5 (5 µg/mL) induced more SA-β-Gal activity (an indicator of cellular senescence) than did the combination of L1 (30 µg/mL) + V1 (5 µg/mL) (Figure 4, *n* = 4 per group; *p* < 0.001 vs. PBS-treated control). This finding supports that L5 + V5 may affect vessel endothelial senescence, which is a risk factor of hard CHD.

### 3.4. Endogenous Levels of Electronegative VLDL and LDL Are Increased in the Serum of ApoE^−/−^ Mice 

**Figure 5:**
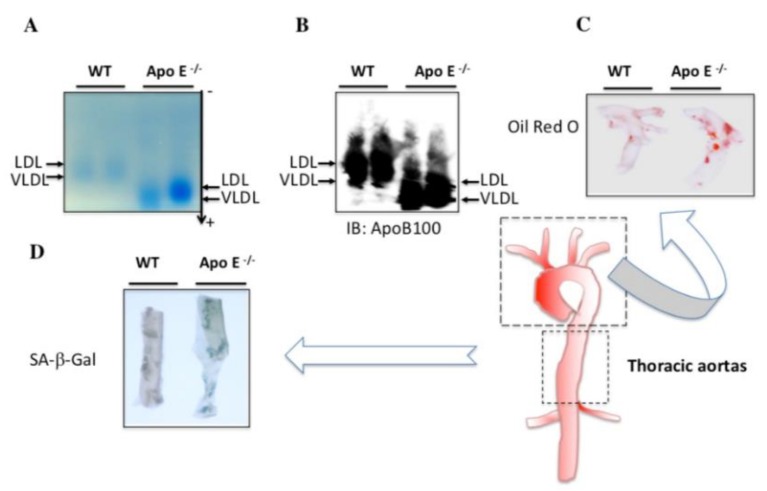
Effect of endogenous L5 and V5 on cellular senescence in vivo. Agarose gel electrophoresis (**A**) and Western blot analysis (**B**) with anti-ApoB100 show the difference between blood serum from wild-type (WT) and ApoE^−/−^ mice fed with a high-fat diet for 15 weeks. Oil Red O (**C**) and senescence-associated β-galactosidase (SA-β-Gal) (**D**) staining of aortas from WT and ApoE^−/−^ mice fed with a high-fat diet. WT (B57CL/6) mice were used as controls.

To validate the prosenescent and proatherogenic properties of electronegative VLDL and LDL in vivo, we conducted experiments with ApoE^−/−^ mice, an atherosclerosis-prone animal model [27]. Endogenous electronegative VLDL and LDL levels were higher in the sera of ApoE^−/−^ mice fed with a high-fat diet than in those of WT mice (Figure 5A,B). Histopathologic analysis of thoracic aortas with Oil Red O and SA-β-Gal staining showed markedly greater lipid accumulation and endothelial cell senescence in ApoE^−/−^ mice than in WT mice (Figure 5C,D). Our in vivo findings support our in vitro findings showing that electronegative VLDL and LDL promote vascular endothelial senescence.

## 4. Discussion

In our study, we showed that the combined amount of plasma L5 + V5 is significantly associated with the adjusted 10-year risk of hard CHD. Our analyses also showed a positive correlation between age and combined L5 + V5 level, suggesting that the senescence response could be affected by increased L5 + V5 levels and that the elevation of L5 + V5 levels may induce senescence of the arterial endothelium. In HAECs, L5, V5, and L5 + V5 induced significantly more SA-β-Gal activity than did L1, V1, or L1 + V1. Furthermore, our in vivo studies in ApoE^−/−^ and WT mice fed with a high-fat diet showed that plasma LDL and VLDL from the ApoE^−/−^ group had significantly greater electrophoretic mobility than did WT counterparts. The increased electronegativity of LDL and VLDL in ApoE^−/−^ mice was also accompanied by increased lipid accumulation and cellular senescence in the aorta. These findings suggest that changes in lipoprotein content and components may increase the risk of CHD and other related comorbidities. 

Aging is a physiologic process associated with increased cardiovascular morbidity and mortality, even in the absence of known cardiovascular risk factors [6]. Vascular cells from human atherosclerotic plaque have shown impaired growth in vitro and underwent senescence earlier than vascular cells from normal vessels [28]. Previously, human atherosclerotic lesions, which contain endothelial cells and vascular smooth muscle cells, were shown to exhibit morphologic features of senescence [29]. Furthermore, Fenton and colleagues showed the presence of SA-β-Gal–positive vascular cells in injured rabbit carotid arteries [30]. When we treated HAECs with a subapoptotic level of L5 (30 µg/mL) and V5 (5 µg/ml), no obvious cellular apoptosis was observed. However, cellular senescence developed after HAECs were continuously incubated with V5 and L5 for 3 days, indicating that the exposure of ECs to a low concentration of V5 and L5 causes endothelial senescence and atherosclerotic change. In addition, ApoE-deficient mice have been shown to develop severe hypercholesterolemia most likely caused by the delayed clearance of large atherogenic particles from the circulation [31]. In our in vivo study, we observed the presence of endogenous electronegative VLDL and LDL in the plasma of ApoE^−/−^ mice fed with high-fat diet. In addition, the electronegativity of LDL and VLDL isolated from ApoE^−/−^ mice was higher than that of WT mice. Furthermore, histopathologic analysis with Oil Red O and SA-β-Gal staining of the thoracic aorta showed significantly more lipid accumulation and endothelial senescence in ApoE^−/−^ mice than in WT mice. Therefore, our findings support a cause-and-effect relationship between V5 and L5 and vascular endothelial senescence. Notably, recent studies have shown that V5 induces reactive oxygen species (ROS) production and that L5 promotes mitochondrial free-radical production and activates the DNA damage response to induce premature vascular endothelial senescence [14,32]. Thus, the possibility remains that ROS production is the underlying mechanism of aging caused by L5 and V5.

Our study has limitations that should be addressed. One important limitation was the cross-sectional study design used to examine the association between CHD risk and combined L5 and V5 levels among our study participants. Moreover, we estimated CHD risk according to the Framingham risk score because we did not obtain prospective follow-up data from these participants regarding CHD events. Although our findings show a significant association between CHD risk and combined L5 and V5 levels, large population-based studies are warranted that include long-term follow-up or longitudinal observations about how L5 and V5 contribute to CHD. In addition, further investigation is needed to evaluate the potential effects of other related factors such as medication use. Because we used questionnaires to collect information such as demographic data, a Neyman bias or other confounding factors may have affected the results of our study. Although we believe our questionnaire was objectively and clearly written, the possibility remains that questionnaire answers may have lacked accuracy, in turn minimizing or maximizing the effects of certain variables. In addition, although SA-β-Gal activity may not be a strict biomarker of senescence, a growing body of evidence has suggested that cellular senescence may contribute to the pathogenesis of vascular aging. In addition, human endothelial cells express telomerase, which is drastically activated by mitogenic stimuli via a protein kinase C–dependent pathway, leading to telomere shortening and cellular senescence [33]. Thus, the possibility remains that atherogenic stimuli increase cell turnover at sites of atherosclerosis, thereby promoting telomere shortening and potentially activating certain proliferative signals that may induce senescence independent from telomere shortening. Moreover, oxidative stress and DNA damage may increase vascular cell senescence and further promote atherogenesis [34]. Therefore, the mechanism by which L5 and V5 induce endothelial cell senescence may involve telomere shortening and DNA damage. 

## 5. Conclusions

In summary, we identified increased levels of L5 + V5 in our group of study participants with high CHD risk, indicating that the electronegativity of L5 and V5 may be important factors influencing hard CHD risk in these individuals. Moreover, we found that risk factors of hard CHD significantly correlated with the total amount of L5 + V5. In cell experiments, we also showed an increased number of cells with positive SA-β-Gal staining after treatment with L5 + V5. Importantly, we observed marked lipid accumulation and endothelial senescence in ApoE^−/−^ mice fed with a high-fat diet, supporting a causal relationship between electronegative VLDL + LDL and vascular senescence. Collectively, this evidence suggests that L5 + V5 may contribute to the overall pathophysiology of CHD through their effects on vessel endothelial senescence, lipoprotein content, and lipoprotein constituents. Future prospective clinical studies are warranted to identify a causal relationship between L5 + V5 and the progression of CHD.

## Figures and Tables

**Table 1:** Characteristics of study participants with high or low hard CHD risk.

	Low Hard CHD Risk (*n* = 17) ^c^	High Hard CHD Risk (*n* = 16) ^c^	*p*-Value ^d^
Age (years)	44.9 ± 6.8	49.3 ± 7.4	0.46
Gender (men: women)	4:13	9:7	0.06
Diabetes mellitus drug treatment	2/17	2/16	0.94
Hypertension drug treatment	6/17	10/16	0.12
Metabolic syndrome	7/17	13/16	0.02
Waist circumference (cm)	97.1 ± 21.9	110.8 ± 11.8	0.17
Body mass index (kg/m^2^)	30.2 ± 8.4	35.1 ± 5.5	0.27
Systolic blood pressure (mmHg)	115.1 ± 21.9	138 ± 11.6	0.01
Diastolic blood pressure (mmHg)	74.5 ± 13.4	81.6 ± 11.5	0.03
Pulse pressure (mmHg) ^a^	40.6 ± 11.8	56.4 ± 11.8	0.03
Mean arterial pressure (mmHg)	88 ± 15.8	100.4 ± 10.1	0.01
Fasting plasma glucose (mg/dL)	94.9 ± 18.4	119.4 ± 40.1	0.05
Total cholesterol (mg/dL)	157.9 ± 29.3	224.1 ± 27.9	<0.001
Triglyceride (mg/dL)	96.4 ± 52	167.3 ± 64.4	0.02
HDL (mg/dL)	48.1 ± 12.2	47.5 ± 10.5	0.75
LDL (mg/dL)	90.6 ± 25.7	143.1 ± 27.6	<0.001
VLDL (mg/dL)	19.3 ± 10.4	33.5 ± 12.9	0.02
LDL/HDL ratio	2 ± 0.6	3.1 ± 0.9	0.003
L5 (mg/dL)	9.2 ± 10.5	27.9 ± 21.8	0.01
V5 (mg/dL)	7.7 ± 6.2	11.5 ± 8.3	0.46
L5 and V5 (mg/dL)	16.9 ± 14.8	39.4 ± 22	0.01
Hard CHD risk (%) ^b^	2.4 ± 2.5	15.3 ± 9.2	<0.001
Average hard CHD risk at the age (%) ^b^	3.4 ± 2.4	6.5 ± 3.5	0.07
Adjusted hard CHD risk ^b^	0.7 ± 0.3	2.4 ± 0.8	<0.001

HDL: high-density lipoprotein; LDL: low-density lipoprotein; VLDL: very low-density lipoprotein; hard CHD: hard coronary heart disease (myocardial infarction, coronary death, or stroke). ^a^ Pulse pressure is equal to systolic blood pressure minus diastolic blood pressure. ^b^ Hard CHD risk is the 10-year risk of myocardial infarction, coronary death, or stroke according to the Framingham risk score. Average hard CHD risk at the age is the average hard CHD risk at the same age derived from the Framingham Heart Study of a predominantly Caucasian population in Massachusetts, USA. Adjusted hard CHD risk means hard CHD risk/average hard CHD risk at the age. ^c^ Data are expressed as the mean ± standard deviation or as a ratio. ^d^ The *p*-value was calculated by using the Mann-Whitney U test, excluding gender, hypertension drug treatment, and metabolic syndrome variables, which were subjected to the Chi-square test, and the diabetes mellitus drug treatment variable, which was subjected to the Fisher exact test.
